# The effect of microdosimetric ^12^C^6+^ heavy ion irradiation and Mg^2+^ on canthaxanthin production in a novel strain of *Dietzia natronolimnaea*

**DOI:** 10.1186/1471-2180-13-213

**Published:** 2013-09-28

**Authors:** Xiang Zhou, Jia-Rong Xie, Lei Tao, Zhi-Jun Xin, Feng-Wu Zhao, Xi-Hong Lu, Mei-Rong Zhao, Liang Wang, Jian-Ping Liang

**Affiliations:** 1 Institute of Modern Physics, Chinese Academy of Sciences, 509 Nanchang Rd, Lanzhou, Gansu 730000, P.R. China; 2 China Pharmaceutical University, #24 Tongjiaxiang, Nanjing 210009, P.R. China; 3 Lanzhou Vocational Technical College, 37 Liusha Rd, Lanzhou, Gansu 730070, P.R. China

**Keywords:** *D. natronolimnaea* svgcc1.2736, Microdosimetric, ^12^C^6+^-ions, Irradiation, Productivity, Canthaxanthin, Response surface methodology

## Abstract

**Background:**

*Dietzia natronolimnaea* is one of the most important bacterial bioresources for high efficiency canthaxanthin production. It produces the robust and stable pigment canthaxanthin, which is of special interest for the development of integrated biorefineries. Mutagenesis employing ^12^C^6+^ irradiation is a novel technique commonly used to improve microorganism productivity. This study presents a promising route to obtaining the highest feasible levels of biomass dry weight (BDW), and total canthaxanthin by using a microdosimetric model of ^12^C^6+^ irradiation mutation in combination with the optimization of nutrient medium components.

**Results:**

This work characterized the rate of both lethal and non-lethal dose mutations for ^12^C^6+^ irradiation and the microdosimetric kinetic model using the model organism, *D. natronolimnaea* svgcc1.2736. Irradiation with ^12^C^6+^ ions resulted in enhanced production of canthaxanthin, and is therefore an effective method for strain improvement of *D. natronolimnaea* svgcc1.2736. Based on these results an optimal dose of 0.5–4.5 Gy, Linear energy transfer (LET) of 80 keV μm^-1^and energy of 60 MeV u^-1^ for ^12^C^6+^ irradiation are ideal for optimum and specific production of canthaxanthin in the bacterium. Second-order empirical calculations displaying high *R-squared* (0.996) values between the responses and independent variables were derived from validation experiments using response surface methodology. The highest canthaxanthin yield (8.14 mg) was obtained with an optimized growth medium containing 21.5 g L^-1^ D-glucose, 23.5 g L^-1^ mannose and 25 ppm Mg^2+^ in 1 L with an irradiation dose of 4.5 Gy.

**Conclusions:**

The microdosimetric ^12^C^6+^ irradiation model was an effective mutagenic technique for the strain improvement of *D. natronolimnaea* svgcc1.2736 specifically for enhanced canthaxanthin production. At the very least, random mutagenesis methods using ^12^C^6+^ions can be used as a first step in a combined approach with long-term continuous fermentation processes. Central composite design-response surface methodologies (CCD-RSM) were carried out to optimize the conditions for canthaxanthin yield. It was discovered D-glucose, Mg^2+^ and mannose have significant influence on canthaxanthin biosynthesis and growth of the mutant strain.

## Background

Microorganisms, because of their phenomenal biodiversity, are a rich natural resource of many biologically active compounds such as proteins, polyunsaturated fatty acids, pigments and polysaccharides [1,2]. Metabolites produced by microorganisms often display high biological activities and their potential health benefits make them valuable ingredients in nutraceuticals, cosmetics and the food industry [3,4]. Moreover, investigations related to the search for new bioactive compounds from industrially important microbial strains are of continued importance because of the high potential economic value of these metabolites [5,6].

Demand for carotenoid (CT) pigments has been growing annually at a rate of 3.1% and is a market predicted to reach at least US$ 1.17 billion in value by 2012 as consumers continue to look for natural alternatives. Among them, canthaxanthin (CX) is used extensively in the food, fishery, cosmetic, and pharmaceutical industries [7,8]. *D. natronolimnaea* is one of the most important sources for the microbial production of CX from a commercial and industrial point of view [9,10]. To meet the growing demand of CX, a cost effective scaling-up of the industrial process is imperative [11]. In conventional methodology, nutritional factors and others necessary for growth of the microorganism are optimized by changing one at a time while keeping all others constant. [12]. This approach is the simplest to implement, and primarily helps in selection of significant parameters affecting the CX yield [13]. Retrospective techniques are not only time restrictive, but also ignore any effects that interaction among various biophysical and nutritional parameters may have [14]. It is necessary to optimize the conditions for CX-producing mutant strains to explore their industrial potential.

Optimization of microbial strains for the overproduction of industrial products has been the hallmark of all commercial bioderived production processes [15]. Traditionally, improvement of bioactive compound yields in wild-type strains has been achieved through ultraviolet (UV) mutagenesis, selection of naturally occurring mutants, or genetic recombination.

In recent years, the term irradiation technology has also been used to refer to novel techniques such as X-rays, ionizing irradiation, and heavy-ion irradiation. Heavy-ion beam irradiation is a type of high linear energy transfer (LET) irradiation that bombards the target with higher energy. Such irradiation usually relies on different doses of irradiation to kill the vast majority of the bacterial cells [16-19]. Following irradiation, the surviving microbes may often contain one or more mutations. For a very small percentage of the survivors the mutation may lead to an improved ability to produce a specific metabolite. Irradiation of bacteria to produce mutant strains that result in the overproduction of primary or secondary metabolites is an intricate process. The successful development of *D. natronolimnaea* svgcc1.2736 mutant strains for example requires knowledge of biophysics, microbiology, cell dynamics and physiology, optimization and control of process parameters, and the design of creative fermentation processes [20-22].

The production of microbial CX is generally carried out through fermentation processes. Such processes provide an excellent system for the large-scale production of carotenoids in general because of their ease of manipulation [23,24]. *D. natronolimnaea* svgcc1.2736 strains have an advantage over other natural bioresources, as the fermentation process can be easily controlled to achieve higher growth rates and greater cell density without infringing on production constraints such as space and time. Studies have shown that maximum production potential of a microbial species can be induced using a number of different approaches. These include supplementation of carotenoid stimulating factor to support enzymes involved in the biosynthetic pathways, empirical optimization of environmental culture conditions through statistical experimental designs, use of stirrer fermenters to boost continuous production of cells in suspension, use of immobilized cell fermenters, screening and selection of optimal procedures for separation, purification, and membrane processing, and the preparation of mutants necessary for genetic engineering and gene expression techniques [25-27].

Detailed measurements of carotenoid and CX levels produced by *D. natronolimnaea* HS-1 mutants have previously been reported by Gharibzahedi, S.M.T., et al. 2012 [9]. The objective of this study therefore, was to apply a microdosimetric kinetic model with Mg^2+^ as a trace element and carry out detailed measurements of CX produced by *D. natronolimnaea* svgcc1.2736 strains using response surface methodology (RSM). This work focuses on the various influencing factors that may be employed to improve *D. natronolimnaea* svgcc1.2736 strains and also addresses the complex problems of media optimization and the fine-tuning of process conditions. Furthermore, this work aimed to explore emerging technologies and optimal media design for tracking mutants displaying enhanced production of microbial CX or other desirable attributes.

## Results and discussion

### Mathematical description of surviving fraction

**Figure 1:**
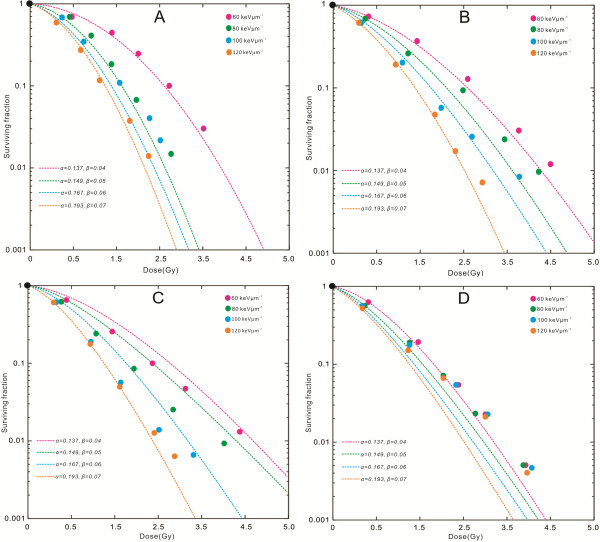
**Survival of normal Dietzia natronolimnaea svgcc1.2736 strains after irradiation by **^**12**^**C**^**6+**^**ion beams of different initial energies and LETs at dose levels of 0.5 to 5 Gy. (A)** Surviving fraction of *D. natronolimnaea* svgcc1.2736 strains after irradiation with 60, 80, 100 and 120 keV/μm (LETs) and 30 MeV/u (energy) ^12^C^6+^-ions are compared. **(B)** Surviving fraction of D. natronolimnaea svgcc1.2736 strains after irradiation with 60, 80, 100 and 120 keV/μm (LETs) and 45 MeV/u (energy) ^12^C^6+^-ions are compared. **(C)** Surviving fraction of *D. natronolimnaea* svgcc1.2736 strains after irradiation with 60, 80, 100 and 120 keV/μm (LETs) and 60 MeV/u (energy) ^12^C^6+^-ions are compared. **(D)** Surviving fraction of D. natronolimnaea svgcc1.2736 strains after irradiation with 60, 80, 100 and 120 keV/μm (LETs) and 90 MeV/u (energy) 12C6*+-*ions are compared.

*D. natronolimnaea* svgcc1.2736 strains were irradiated by four energies: 30 MeV u^-1^, 45 MeV u^-1^, 60 MeV u^-1^ and 90 MeV u^-1^, generated by a ^12^C^6+^ heavy ion accelerator. Initial LET beam energies of the ^12^C^6+^ ions were 60 keV μm^-1^, 80 keV μm^-1^, 100 keV μm^-1^ and 120 keV μm^-1^, respectively. Figure 1 shows survival curves of the strains with different energies and LETs. The survival curves were fitted by a linear quadratic model, which for the four energies gave values of 0.137±0.003 Gy^-1^ and 0.04 Gy^-2^, 0.149±0.005 Gy^-1^ and 0.05 Gy^-2^, and 0.167±0.006 Gy^-1^ and 0.193±0.007 Gy^-1^ respectively. The essential difference compared with Equation (3) is, that the linear-quadratic approach allows for a finite initial slope to be calculated [28]. The different values correspond to curves obtained from the standard graph and use of Equation (4) [29]. These curves assume the effectiveness towards microdosimetry is completely described by the linear α-term in Equation (4) [30]. Fitting two parameters to the limited survival data of these strains would cause large errors because of anticorrelation between *α* and *β* values [31]. For this reason only the *α* value was fitted with a constant *β* value. This is analogous to the microdosimetric kinetic model (MKM) used to calculate relative biological effectiveness (RBE) values. Equation (5) is a general formula used in the local effect model [32]; it does not rely on any particular representation of the photon dose response curve [33]. The formula can be applied even if only numerical values of *S(D)* are available [34]. For practical reasons, however, a linear-quadratic approach for the low-LET dose response curve is generally used [35].

Interpretation of the parameter fitting RBE/LET dependencies in this study indicating an increased RBE is not unique for carbon ions of charged particle radiation. The RBE values derived from the survival curves support the known dependence of RBE on LET, particle species and dose [36]. For ^12^C^6+^ ions, the transportation safety technologies (TST)-calculated RBE/LET dependencies gradually increase with increasing LET until they reach a maximum value, after which they slowly decrease [37]. The dependencies rely strongly on the particular physical characteristics of the ion beam determined for example by the energy and LET of the particles under consideration [38]. This is demonstrated in Figure 1 (A, B, C and D), where survival curves of *D. natronolimnaea* svgcc1.2736 cells after irradiation with 60, 80, 100 and 120 keV μm^-1^ (LET) and 30, 45, 60 and 90 MeV u^-1^ (energies) ^12^C^6+^ ions are compared. Each survival curve has been constructed using a linear-quadratic model [39]. RBE decreases with increasing particle energy [40], and the same increased ionization density should hold true for all cell types [41]. Because the ^12^C^6+^ ions have a higher energy for any given LET, lower energy density and thus lower RBE result. One must bear in mind, however, that high ionization densities will lead to more extensive damage that is more difficult to repair. Cellular defects arising from damage repair may not necessarily translate into increased effectiveness because even simple damage is not always repairable by the cell [42,43]. Survival data of the *D. natronolimnaea* svgcc1.2736 cells were plotted using a logarithmic function of the surviving fraction versus dose. For comparison purposes the curves were represented mathematically, based on hypothetical models for the mechanisms associated with lethality. Interpretation of the shape of the survival curve is still in question, as is the best way to mathematically present these types of data sets. The interpretation of the shape of the cell survival curve is still debated, as is the best way to fit these types of data mathematically. As already indicated in Figure 1A-D, after reaching a maximum at 120 keV μm^-1^ surviving fraction not further increases, but instead decreases towards higher dose values. For the ^12^C^6+^ heavy ion irradiation (A dose of ≥2.5 Gy for ≥45 MeV u^-1^) surviving fraction values as low as 1% are observed. The strain cells survival as a function of dose follows almost exponential behaviour, and thus survival curves are generally shown in Figure 1A-D. The most prominent feature of most survival curves is thus the deviation from such a simple curve; namely, the dose response curve typically shows a shoulder (Figure 1). For the *D. natronolimnaea* strain cell types, survival curves start with a moderate slope, and with increasing energy and dose, the slope correspondingly increases. Therefore, the efficiency per energy and dose increment increases as well. This can be understood in terms of the effectively of radiation induced mutations. At low energies and doses, only a few mutations are induced with a large spatial separation, and a considerable fraction of these mutations can be irradiated effectively. In contrast, at high energies and doses, the density of mutations increases, leading to an interaction of mutations and thus a reduced surviving fraction.

### Effect of different ^12^C^6+^ irradiation on cell growth

**Figure 2:**
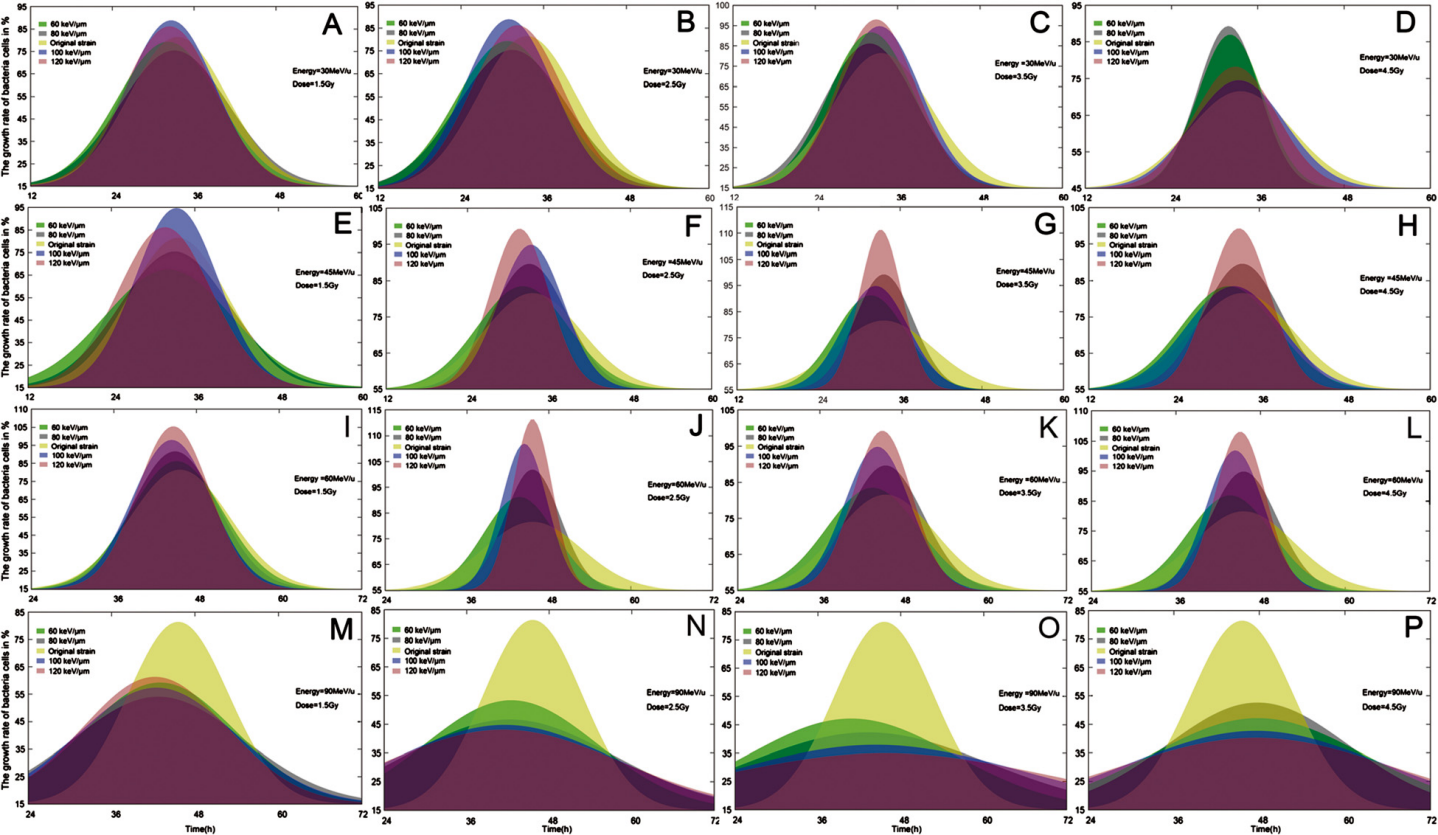
^**12**^**C**^**6+**^**-ions of different parameters irradiation level and its effect on the growth rate of *D. natronolimnaea *smgcc1.2736 strains cells in %. (A-D) **^12^C^6+^-ions were accelerated up to 30 MeV/u, and their LETs were 60, 80, 100 and 120 keV/μm, with a dose rate of 0.5-1.5Gy. **(E-H) **^12^C^6+^-ions were accelerated up to 45 MeV/u, and their LETs were 60, 80, 100 and 120 keV/μm, with a dose rate of 0.5-1.5Gy. **(I-L) **^12^C^6+^-ions were accelerated up to 60 MeV/u, and their LETs were 60, 80, 100 and 120 keV/μm, with a dose rate of 0.5-1.5 Gy. **(M-P) **^12^C^6+^-ions were accelerated up to 90 MeV/u, and their LETs were 60, 80, 100 and 120 keV/μm, with a dose rate of 0.5-1.5 Gy.

Following irradiation, serial dilutions of the cell suspension to be tested were prepared. Ten microliters of each dilution was inoculated into a 96-well plate containing 180 μL of the growth medium. For each dilution 10 replicates were prepared. Plates were incubated at 27°C for 96 hours as previously described. The cell concentration was determined using the Reed and Muench method [44]. In each individual experiment, a cell culture was divided into aliquots and subjected to a predetermined set of irradiation doses, including no irradiation exposure. The aliquots were diluted in growth medium immediately after irradiation and plated in duplicate or triplicate [45]. For each experiment, the multiple platings of unirradiated (0 Gy) aliquots were counted and averaged to give the initial cell density in CFU mL^-1^. This value represented 85–100% cell growth of the strain and was used as a base level comparison for all irradiated aliquots of the same culture. Optical density (OD) measurement at 600 nm was used to monitor cell growth. Wherever necessary, samples were diluted to a final OD value lower than 0.3 [46]. For all irradiation conditions examined, the concentrations of viable cells increased in an exponential fashion, followed by the typical stationary and death phases (Figure 2). Microdosimetry using ^12^C^6+^ ions for the mutagenesis of *D. natronolimnaea* svgcc1.2736 strains clearly shows an exponential decrease in the growth rate from 85% (0 Gy), to approximately 27% (LET 120 keV μm^-1^, energy 90 MeV u^-1^ and a dose of 3.5 Gy) (Figure 2O). 113% (Figure 2J) at LETs (120 keV μm^-1^), energies (60 MeV u^-1^) and dose (2.5 Gy), to about 111% (Figure 2G) at LETs (120 keV μm^-1^), energies (45 MeV u^-1^) and dose (3.5 Gy), to about 97% ( Figure 2C) at LETs (120 keV μm^-1^), energies (30 MeV u^-1^) and dose (3.5 Gy). Interestingly, many survivors of the high-energy irradiation displayed a significant delay in growth and required extended incubation times to allow formation of measurable sized colonies. Many of the low-energy survivors, however, displayed significant growth acceleration and therefore required shorter incubation times to form macroscopic colonies [47]. Figure 2E–H shows the maximum growth rate (98–111%) for 28 h obtained using irradiation parameters, 60 MeV u^-1^ (energy), 60–120 keV μm^-1^ (LET) and 1.5–4.5 Gy (dose). Figure 2M–P presents the minimum growth rate (27–58%) for 42 h obtained using the irradiation parameters, 90 MeV u^-1^ (energy), 60–120 keV μm^-1^ (LET), and 1.5–4.5 Gy (dose). These data suggest that the cellular growth rate of the *D. natronolimnaea* svgcc1.2736 strain is dependent on the irradiation energy of the ^12^C^6+^ions. Significant differences in the effects of ^12^C^6+^ ions at the same doses were also observed. This suggests a strong dependence of low-dose effects on LET (Figure 2I-L).

### *Effect of irradiation dose on productivity of D. natronolimnaea svgcc1.2736*

**Figure 3:**
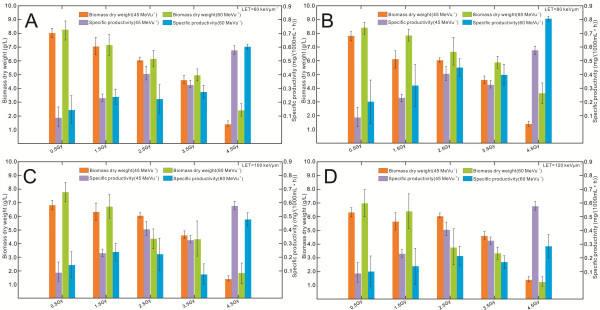
**Influence of different irradiation dose (energy=45,60 MeV/u) on the *D. natronolimnaea*svgcc1.2736 strains biomass dry weight and productivity. (A)** LET for 60 keV/μm post-irradiation, 72 hours of cultivation illustrating the effect of biomass dry weight and specific productivity. **(B)** LET for 80 keV/μm post-irradiation, 72 hours of cultivation illustrating the effect of biomass dry weight and specific productivity. **(C)** LET for 100 keV/μm post-irradiation, 72 hours of cultivation illustrating the effect of biomass dry weight and specific productivity. **(D)** LET for 120 keV/μm post-irradiation, 72 hours of cultivation illustrating the effect of biomass dry weight and specific productivity.

Different irradiation doses showed a notable affect on the growth rate and conidia aggregation in *D. natronolimnaea* svgcc1.2736. CX production in 1 L cultures of *D. natronolimnaea* svgcc1.2736 mutants was, shown to be sensitive to irradiation dose (Figure 3). Overall, for CX producing strains of *D. natronolimnaea* svgcc1.2736 mutants, increasing the irradiation dose from the standard 0.5 to 4.5 Gy led to a considerable decline in dry cell weight (BDW), from around 8.71 ±0.04 to 2.23 ±0.06 g L^-1^, respectively. The CX yield, however, showed an almost two-fold increase from 8 ±0.9 to 12 ±0.2 mg L^-1^. To find the optimal ^12^C^6+^ irradiation dose for the process, a considerable amount of cell culture was carried out using similar irradiation experiments. Figure 3A shows that up to a dose of 4.5 Gy irradiation, the *D. natronolimnaea* svgcc1.2736 strains productivity increases by almost six-fold. Optimal production of 0.81 mg L^-1^ h^-1^ was detected at a irradiation dose of approximately 4.5 Gy at an 80 keV μm^-1^ LET and 60 MeV u^-1^ energy level (Figure 3B). In contrast, ^12^C^6+^ irradiation with a LET of more than 100 keV μm^-1^, and energy level of greater than 45 MeV u^-1^ reduced the rate of production (Figure 3D). ^12^C^6+^ irradiation with LET (80 keV μm^-1^), energy (60 MeV u^-1^) and dose (1.5 Gy) led to perfect mycelial growth (Figure 3A). The increased irradiation dose of ^12^C^6+^ however led to a decrease in biomass in this strain (Figure 3). Figure 3B depicts the BDW and productivity of the strains with respect to different energy (45 and 60 MeV u^-1^) versus an irradiation dose with a LET of 80 keV μm^-1^. Productivity increased with increasing irradiation dose and energy up to 4.5 Gy and 60 MeV u^-1^ respectively. The specific productivity decreased at radiation doses less than 1.5 Gy. In contrast, the BDW yield decreased with increasing irradiation dose and energy up to 4.5 Gy and 60 MeV u^-1^ respectively. Figure 3D depicts the BDW and productivity of the strains with respect to different energy (45, and 60 MeV u^-1^) versus an irradiation dose at a LET of 120 keV μm^-1^. As the radiation dose (0.5–4.5 Gy) and energy (60 MeV μm^-1^) increased, the BDW yields decreased from 7.20 to 1.26 g L^-1^. However, the maximum specific productivity was measured at just 0.27 mg L^-1^ h^-1^. Further increases in radiation doses resulted in decreased BDW and specific productivity. The wild type strain of *D. natronolimnaea* svgcc1.2736 was used in this study to substantiate the findings made with irradiated strains. Just 20 cell cultures using wild type strains were carried out. This resulted in the wild type strains displaying a higher standard deviation (Figure 3A–D) compared with the standard deviation of the 40 irradiated strains. Throughout the study, it was observed that the BDW declined concomitantly with increasing bacterial specific productivity. The BDW dropped to its minimum when microorganism specific productivity peaked. From our findings it is evident that irradiation doses (120 keV μm^-1^ of LET and 60 MeV u^-1^ of energy level) greater than 4.5 Gy can both damage cells and/or change cell morphology, which leads to reduced CX yields. The optimal LET, Energy and irradiation dose for the non-lethal induction of point mutations by ^12^C^6+^ ions (LET=80 keV μm^-1^, energy=60 MeV u^-1^ and dose=0.5–4.5 Gy) are also ideal for maximising CX specific productivity in *D. natronolimnaea* svgcc1.2736.

### Statistical evaluation and optimization of factors affecting productivity by RSM

Canthaxanthin production is generally carried out through fermentation processes [48]. Because of their ease of manipulation microorganisms provide an excellent system that facilitates large-scale production of CX. Optimization of conditions favouring CX production in irradiated strains is necessary to explore their industrial possibilities [49]. This can be achieved through RSM, a type of modelling used to study the effects of simultaneous variation of several factors [50]. RSM studies help to determine the accurate optimum values of test variables on the basis of a limited number of experiments [51]. Carbon source was examined in a supplemented basal medium containing, D-glucose, maltose, mannose, lactose, galactose and glycerol. Preliminary investigations demonstrated that D-glucose and mannose were significant carbon sources for production of CX (data not shown). Trace elements such as Cu^2+^, Fe^3+^, Zn^2+^ Mn^2+^ and Mg^2+^ act as cofactors for several enzymes involved in the biosynthesis of carotenoids, and at certain concentrations, can improve metabolite production [52,53]. In addition, it has been reported that supplementation of the growth medium with various ions (Cu^2+^, Fe^2+^, Zn^2+^, Mn^2+^) improved carotenoid production by various yeast strains including *Rhodotorula glutinis*[54,55]. It has also been reported that the rate of carotenogenesis in the fungus *Blakeslea trispora* was significantly elevated in the presence of Fe^3+^, Mg^2+^ and Cu^2+^ ions. A preliminary investigation demonstrated that divalent ions including Mg^2+^, boron, cobalt, iron, manganese, molybdenum, selenium and vanadium had the highest effect on CX biosynthesis in *D. natronolimnaea* svgcc1.2736 mutants (data not shown). RSM was used to evaluate the effect of four variables on the growth and CX production of *D. natronolimnaea* svgcc1.2736 ^12^C^6+^ irradiation mutants. These were D-glucose content (12.5–25 g L^-1^, A), Mg^2+^ concentration (15–40 ppm, B), mannose content (6.75–25 g L^-1^, C) and irradiation dose (0.5–4.5 Gy, LET=80 keV μm^-1^ and energy=60 MeV u^-1^, D). Where Sqrt is equal to CX production, the model incorporating the four variables (Equation 1) is as follows:

(1) $$\begin{array}{l} \mathrm{Sqrt}=\varpi_{0}+\varpi_{1}A+\varpi_{2}B+\varpi_{3}C+\varpi_{4}D+\varpi_{11}A^{2}\\ \;+\varpi_{22}B^{2}+\varpi_{33}C^{2}+\varpi_{44}D^{2}+\varpi_{12}\mathrm{AB}+\varpi_{13}\mathrm{AC}\\ \;+\varpi_{14}\mathrm{AD}+\varpi_{23}\mathrm{BC}+\varpi_{24}\mathrm{BD}+\varpi_{34}\mathrm{CD} \end{array}$$

Based on central composite design (CCD), 30 treatments, each at three different levels (−1.25, 0 and +1.25) were carried out. Experiments were randomized to minimize the effects of unexplained variability in the observed responses due to extraneous factors [56,57]. These preliminary studies showed that upon addition of the four growth factors (at concentrations specified above) to the culture medium, desirable amounts of BDW and CX were produced by the mutant strain (Table 1).

**Table 1:** **Full factorial CCD design matrix of four variables with the observed responses for CX produced by the bacterium *D. natronolimnaea *svgcc1.2736**

**Standard**	**D-glucose**	**Mg**^**2+**^**(MgSO**_**4**_**)**	**Mannose**	**Dose**	**CX(mg/1000 mL)**		**BDW(g/1000 mL)**	
**order**	**(g/L)**	**(ppm)**	**(g/L)**	**(Gy)**				
	**Factor A**	**Factor B**	**Factor C**	**Factor D**	**Observed**	**Predicted**	**Observed**	**Predicted**
1	12.5	15	6.75	3.5	4.78±0.07	4.66	6.47±0.17	6.39
2	25	15	6.75	3.5	5.63±0.09	5.58	8.73±0.12	8.59
3	12.5	40	6.75	3.5	4.89±0.05	4.76	6.81±0.13	6.75
4	25	40	6.75	3.5	5.61±0.02	5.53	7.63±0.09	7.53
5	17.5	25	25	0.5	7.12±0.05	7.09	7.94±0.05	7.86
6	17.5	25	6.75	0.5	6.34±0.03	6.24	11.35±0.07	11.03
7	17.5	25	25	4.5	6.78±0.11	6.59	9.63±0.09	9.34
8	17.5	25	6.75	4.5	6.89±0.08	6.74	9.24±0.05	9.12
9	25	25	13.75	0.5	7.23±0.12	7.11	6.53±0.06	6.29
10	12.5	25	13.75	0.5	8.13±0.07	8.08	8.96±0.10	8.78
11	25	25	13.75	4.5	5.36±0.04	5.24	9.65±0.12	9.47
12	12.5	25	13.75	4.5	7.21±0.10	7.11	9.54±0.07	9.35
13	17.5	15	6.75	4.5	8.47±0.12	8.37	8.42±0.05	8.33
14	17.5	40	6.75	3.5	7.47±0.07	7.27	8.76±0.03	8.67
15	17.5	15	25	3.5	6.21±0.09	6.09	7.35±0.12	7.22
16	17.5	40	25	3.5	7.21±0.07	7.14	6.77±0.15	6.59
17	12.5	25	13.75	3.5	6.34±0.02	6.11	6.35±0.09	6.24
18	25	25	13.75	3.5	5.36±0.03	5.22	7.23±0.06	7.18
19	12.5	25	25	3.5	6.31±0.12	6.18	7.02±0.05	6.99
20	17.5	25	25	3.5	6.24±0.05	6.09	6.64±0.13	6.48
21	17.5	15	25	0.5	5.37±0.07	5.27	7.95±0.15	7.66
22	17.5	40	13.75	0.5	5.89±0.13	5.63	8.85±0.04	8.77
23	17.5	15	13.75	4.5	5.35±0.04	5.27	9.06±0.08	8.97
24	17.5	40	13.75	4.5	6.86±0.08	6.63	7.12±0.06	7.09
25	17.5	25	13.75	3.5	8.95±0.02	8.95	10.53±0.12	10.53
26	17.5	25	13.75	3.5	8.95±0.02	8.95	10.53±0.09	10.53
27	17.5	25	13.75	3.5	8.95±0.03	8.95	10.53±0.10	10.53
28	17.5	25	13.75	3.5	8.95±0.01	8.95	10.53±0.08	10.53
29	17.5	25	13.75	3.5	8.95±0.03	8.95	10.53±0.07	10.53
30	17.5	25	13.75	3.5	8.95±0.01	8.95	10.53±0.05	10.53

The statistical significance of the model Equation (1) was determined by Fishers test value. The degree of variance illustrated by the model is given by the *R* squared value [58,59]. The statistical treatment combinations of the process parameters along with the BDW concentrations (g L^-1^) and CX production (mg L^-1^) as response variables are listed in Table 1. The regression equation was assessed statistically by ANOVA, the results of which are presented in Table 2. Analysis of variance showed a high degree of significance for CX yield, which is also evident from the Fisher *F* test (*F*_*model*_-1.563E+005 for CX), which gave a very low *p* value in both cases.

**Table 2:** Analysis of ANOVA for response surface quadratic model

**Source**	**Sum of squares**	**DF**	**Mean square**	**F-value**	***P*-value**
Model	1.563E+005	14	21.3725	163.68	<0.0001
A-(D-glucose)	0.4723	1	0.4723	0.0273	<0.0001
B-(MgSO_4_)	1.0347	1	1.0347	0.1654	<0.0001
C-(Mannose)	0.6328	1	0.6328	0.0526	<0.0001
D-(Dose)	1.5634	1	1.5634	0.0127	<0.0001
AB	0.3216	1	0.3216	0.0362	0.2875
AC	0.1478	1	0.1478	0.0168	0.8731
AD	0.2357	1	0.2357	0.0179	0.0002
BC	0.3246	1	0.3246	0.1531	<0.0001
BD	1.7634	1	1.7634	0.9635	<0.0001
CD	2.3564	1	2.3564	0.2238	0.3251
A^2^	0.7532	1	0.7532	0.0736	0.0002
B^2^	1.0478	1	0.0478	0.1398	<0.0001
C^2^	1.6352	1	1.6352	0.1627	<0.0001
D^2^	1.3546	1	1.3546	0.1335	<0.0001
Residual	0.005	14	0.005		
Lack of fit	0.005	10	0.005		
Pure error	0.0001	4	0.0001		
Cor total		1.563E+005			
Standard deviation		0.62		R-squared	0.9963
Mean		62.347		Adjusted R-squared	0.9945
Coefficient of variation (C.V. %)		0.72		Predicted R-squared	0.9333

The *F*- value of 163.68 in Table 2 implies that the model is significant. There is only 0.01% chance that a “model *F*-value” so large could occur due to noise. ANOVA indicated that the linear model terms, D-glucose content (*p* <0.0001), Mg^2+^ concentration (*p* <0.0001), mannose content (*p* <0.0001), dose (*p* <0.0001), and the quadratic terms, D-glucose A^2^ (*p* <0.0002), Mg^2+^ concentration B^2^ (*p* <0.0001), mannose content C^2^ (*p* <0.0001), dose D^2^ (*p* <0.0001) and four interaction terms were significant. ANOVA was used to analyze the responses under different combinations as defined by the design (Table 2). The application of RSM gave rise to the regression Equation (2) for CX production. The quadratic equation specifies an empirical relationship between CX yield and the test variables.

(2) $$\begin{array}{l} \mathrm{Sqrt}=9.2486+0.4723A+1.0347B+0.6328C\\ \;+1.5634D-0.7532A^{2}-1.0478B^{2}-1.6352C^{2}-1.3546D^{2}\\ \;-0.3216\mathrm{AB}+0.1478\mathrm{AC}-0.2357\mathrm{AD}+0.3246\mathrm{BC}\\ \;+1.7634\mathrm{BD}+4.3564\mathrm{CD} \end{array}$$

The ANOVA regression model demonstrated an adjusted coefficient of determination (*R*^*2*^_*adjusted*_) of 0.9945, indicating 99.45% variability in the response could be explained by this model. A very low value of coefficient of variation (C.V., 0.72%) indicates better precision and reliability of the executed experiments. An acceptable precision value of 64.594 was obtained as a measure of the signal-to-noise ratio, with a ratio >3.6 deemed desirable [60-62]. In this case, higher ratio indicates an adequate signal, and also proves that model can be used to navigate the design space [63].

**Figure 4:**
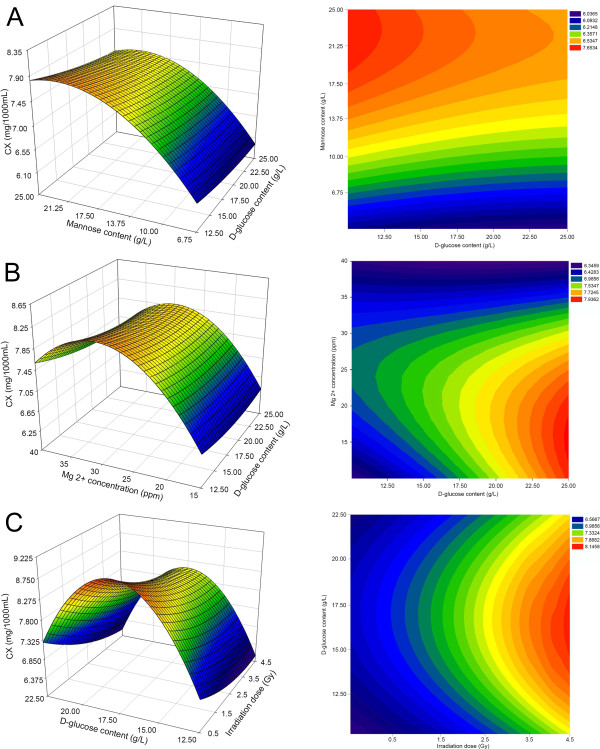
**Response surface curve (Left) and Contour plot (Right) of CX production by *D. natronolimnaea *svgcc1.2736 showing mutual interactions between showing mutual interactions between (A) D-glucose and mannose, (B) D-glucose and Mg**^**2+**^**, (C) **^**12**^**C**^**6+**^**-ions irradiation dose and D-glucose.** Other variables, except for those presented here, were maintained at zero.

Table 2 shows the linear effects of D-glucose content and Mg^2+^ concentration were significant (*p* <0.0001) on the CX produced by *D. natronolimnaea* svgcc1.2736 mutants, whereas mannose content was significant. The quadratic effects of mannose content and Mg^2+^ concentration were significant at the 0.002% level. In Table 2 depicts an interaction between D-glucose and mannose content was not significant. These observations were also substantiated by a highly significant (*p* <0.001) interactive effect between the variables on biomass production. The 3D response surface plots and two dimensional contour plots were used to understand the interaction effects of medium components and optimum concentration of each component required for maximum CX production. In each set, two variables varied within their experimental range, while the other two variables remained constant at zero level. This reveals that variation in the CX value could be explained as a nonlinear function of the D-glucose and mannose content. The most significant (*p* <0.001) effect on CX was shown to be the linear effect of Mg^2+^ concentration, followed by the linear effect of D-glucose content and the quadratic effect of Mg^2+^ concentration, as presented in Table 2. The concentration of Mg^2+^ can therefore significantly influence the production and accumulation of biomass [64]. Mg^2+^ acts as a stimulant by affecting the growth and activity of the microorganism, which in turn leads to a significant improvement in microbial biomass and production of CX [65]. Figure 4A shows the response surface contour plot and 3D plots for the interactive effect of D-glucose and mannose on CX production. It was observed that mutants of *D. natronolimnaea* svgcc1.2736 grown in D-glucose medium and supplemented with 13.5 g L^-1^ mannose showed an increase in CX (7.65 mg L^-1^). However, CX concentration significantly decreased upon further increases in mannose content. This was likely due to inhibition facilitated by sugar concentrations higher than 13.5 g L^-1^[9]. The results suggest that a culture medium containing 23.50 g L^-1^ D-glucose, 11.75 g L^-1^ mannose and 31.16 ppm Mg^2+^ is optimal for obtaining maximum CX production.

Response surface contour and 3D plots were employed to determine the interaction of the independent variables and the optimum levels that have the most significant effect on CX production (Figure 4A–C). Table 2 indicates the quadratic effects of irradiation dose and mannose content significantly (*p* <0.001) influenced the production of CX. Moreover, the interaction between irradiation dose and D-glucose concentration was significant (*p* <0.001). Among the four interaction parameters studied, irradiation dose was the most significant factor to affect the CX obtained from *D. natronolimnaea* svgcc1.2736 mutants. This was followed by the linear effect of D-glucose content and the quadratic effect of mannose content, according to the significance of the regression coefficients in the quadratic polynomial model (Table 2) and slope of the 3D response surface plot (Figure 4B and C). Figure 4B shows that high D-glucose and Mg^2+^concentrations were responsible for the high CX value. The interaction response of D-glucose with Mg^2+^ resulted in an increasing CX yield with increasing D-glucose and Mg^2+^ concentrations up to 17.5 g L^-1^ and 25 ppm, respectively. The CX production increased when Mg^2+^ concentrations >18.5 ppm. The optimal values for D-glucose content and Mg^2+^concentration were 23.5 g L^-1^and 21.5 ppm, respectively. Figure 4C illustrates the interactive effect of D-glucose content (12.5–25 g L^-1^) and irradiation dose (0.5–4.5 Gy) on CX production. It was observed that a combination of both irradiation dose and D-glucose content was solely responsible for achieving a relatively high CX yield of 8.14 mg L^-1^ as predicted by the model. CX production in the bacterial strain, *D. natronolimnaea* svgcc1.2736 could therefore theoretically be increased 1.5 fold from 5.24 to 8.14 mg L^-1^, using mutagenesis. To our knowledge, the maximum CX production by *D. natronolimnaea* strains without the use of cofactors and mutagenic processes was reported at 5.78 mg L^-1 ^[66-69]. The mutant *D. natronolimnaea* svgcc1.2736 strain obtained from ^12^C^6+^ mutagenesis in the presence of a radiation dose of 3.5–4.5 Gy therefore exhibited 64.37% more CX production than the wild type. In comparison, the mutagenesis work of Gharibzahedi *et al.* on the same bacterium reported CX production of 7.10 mg L^-1^.

## Conclusions

Microdosimetry using a ^12^C^6+^ heavy ion irradiation model used parametric models for determination of the optimal doses required for the non-lethal induction of mutations (LET = 80 keV μm^-1^, energy = 60 MeV u^-1^ and dose = 4.5 Gy). Statistical evaluation and response surface methodologies were used to model optimization of CX production from the mutant strain of *D. natronolimnaea* svgcc1.2736. CCD was a key tool for optimizing the components of the nutrient medium. The model was successfully demonstrated by raising the productivity of the mutant *D. natronolimnaea* svgcc1.2736 strain. A 63.37% increase in CX production was evident when nutritional factors (D-glucose content 21.5 g L^-1^, mannose content 23.5 g L ^-1^, Mg^2+^ concentration 25 ppm) and irradiation doses (4.5 Gy) were optimized. At the very least, ^12^C^6+^ random mutagenesis can be used as a first step in a combined approach with continuous fermentation processes. We believe that the data obtained from this work are valuable and should be developed further.

## Methods

### Microorganism and cultivation

The *D. natronolimnaea* strains svgcc1.2736 in this work were obtained from the heavy ion radiation Drug R & D Center at Institute of Modern Physics and selected for polyphasic taxonomical comparison. The bacterium suspension grown in yeast/maltagar (AT medium) that consisted of 0.7 g KH_2_PO_4_; 0.8 g MgSO_4_**·** 7H_2_O; 6 g KNO_3_; 0.03 g FeSO4 **·** 7H_2_O; 0.03 g CaCl_2_**·** 2H_2_O; 0.003 g MnSO_4_**·** nH2O; 0.0006 g ZnSO_4_**·** 7H2O; 15 g agar in 1000 mM NaHCO_3_/ Na_2_CO_3_ buffer (pH=7.25) in deionized water, supplemented with vaporized glucose as the sole carbon source [70]. Every month, single colonies were transferred to a fresh plate, incubated for 3 days, and then maintained under refrigeration at 0–3°C. All cultures were grown in a humidified 90%, air/6% CO_2_ atmosphere at 27°C.

### ^12^C^6+^-ion Irradiations

The ^12^C^6+^-ion irradiations were performed at room temperature and under atmospheric conditions. The details of the irradiation setup are described elsewhere [71]. Briefly, A total spores at a cell density of about 1×10^9^ cells mL^-1^ for each spore line were collected into a multipurpose incubation chamber (100 × 100 mm, Cosmo Bio Co.,Ltd.) and irradiated using a HIRFL cyclotron (Heavy Ion Research Facility in Lanzhou) with a priming dose of 0.5-5 Gy, dose rates were up to 0.1 Gy min^-1^, These ^12^C^6+^-ions were accelerated up to 30 MeV u^-1^, 60 MeV u^-1^, 90 MeV u^-1^ and their LETs were 60, 80, 100 and 120 keV μm^-1^, respectively [72]. After irradiation, part of the frozen (stored in 30% glycerin at −80°C) used in subsequent experiments, while another part of all organisms were grown for an additional 9 h at 27°C and then harvested by centrifugation, resuspended in approximately 150 mL of AT medium and the numbers of spores were counted to determine survival rates.

### Calculation model for survival dose response curve

For ^12^C^6+^-ion radiotherapy in Lanzhou, China, the relative biological effectiveness (RBE)-weighted absorbed dose was defined as a product of the absorbed dose and RBE for *D. natronolimnaea* strains cells death of in vitro. The *D. natronolimnaea* strains cells have been used to determine the RBE in ^12^C^6+^-ion beams as the standard reference cell line. The irradiation ^12^C^6+^-ion beams were designed to effect a 10% survival fraction for the strains cells in the region of the spread-out Bragg peak (SOBP) [73]. The surviving fraction, *S(D)*, was calculated from the lineal energy spectrum by the MKM as follows:

(3) $$S\left(D\right)=S_{0}\left[1-{\left(1-e^{-D/D_{0}}\right)}^{m}\right]$$

Where *D* is the dose, *S* is the survival probability for unirradiated control cells, *D*_*0*_ is related to the steepness of the curve at high doses and m is the target number.

In the modified MKM, the surviving fraction, *S(D)*, of certain cells is calculated with the biological model parameters (α_0_, *β*, *r*_*d*_ and *y*_*0*_); since most cell lines actually show a finite initial slope [74]. This can be better described using the so-called “linear-quadratic” approach, as follows:

(4) $$S\left(D\right)=\exp\;\left[-\left(\alpha_{0}+\frac{\beta }{\rho \pi r_{d}^{2}}y^{*}\right)D-\beta D^{2}\right]$$

(5) $$y^{*}=\frac{y_{0}^{2}\int \left(1-\exp\left(-y^{2}/y_{0}^{2}\right)\right)f\left(y\right)\mathrm{dy}}{\int \mathrm{yf}\left(y\right)\mathrm{dy}}$$

Where *D* is the absorbed dose, is the density of tissue assumed to be *ρ* =1g/cm^3^, *f(y)* is the probability density of lineal energy, *y*, y* represents the saturation-corrected dose-mean lineal energy and *β* is the constant value of 0.05 Gy ^-2^.

### Optimization of media and cultivation parameters

After irradiation, a modified various nutritional with the composition listed as follows (in g L^-1^) was used as the growth medium for all. The *D. natronolimnaea* svgcc1.2736 original strains cultivations: D-glucose 27.0; uridine 0.135; 60 mL L^-1^ saltsolution containing 126 g L^-1^ (NH_4_)_2_SO_4_; 5 g L^-1^ MgSO4 **·** 7H2O; 60 g L^-1^ KH_2_PO_4_; 2 g L^-1^ CaCl_2_**·** 2H_2_O and 0.3 mL L^-1^solution containing trace element: 60 g L^-1^ C_6_H_8_O_7_**·** H_2_O; 60 g L^-1^ ZnSO_4_**·** 7H_2_O; 15 g L^-1^ Fe(NH_4_)_2_(SO_4_)_2_**·** 2H_2_O; 0.9 g L^-1^ Na_2_MoO_4_**·** H_2_O; 1.8 g L^-1^ CuSO_4_; 0.9 g L^-1^ H_3_BO_3_; 0.18 g L^-1^ MnSO_4_**·** H_2_O. The cultivation medium of *D. natronolimnaea* svgcc1.2736 by ^12^C^6+^-ion irradiation, contained per liter 25 g D-glucose as 25 mL saltsolution (6 g L^-1^ NaNO_3_, 0.5 g L^-1^ KCI, 1.5 g L^-1^ KH_2_PO_4_, 0.5 g L^-1^ MgSO_4_**·** 7H_2_O) and 2 mL solution containing trace element (15 mg L^-1^ EDTA, 6.3 mg L^-1^ ZnSO_4_**·** 7H_2_O, 0.09 mg L^-1^ MnCl_2_**·** 4H_2_O, 0.27 mg L^-1^ CuSO_4_**·** 5H_2_O, 1.17 mg L^-1^ CaCl_2_**·** 2H_2_O, 1.5 mg L^-1^ FeSO_4_**·** 7H_2_O, 0.09 mg L^-1^ CoCl_2_**·** 6H_2_O and 0.36 mg L^-1^ (NH_4_)_6_Mo_7_O_24_**·** 4H_2_O). Initial pH of the medium=7.0, shaking speed=180 rpm, temperature=28±3°C and time of incubation=72 h were the physical parameters studied for their effect on bacterial growth and CX production [75]. D-glucose, solution containing trace element and saltsolution were autoclaved separately at 125°C for 25 min and chilled to room temperature prior to mixing and use [76].

### Growth kinetics and biomass concentration

After irradiation, cultures were inoculated with 0.9% (v/v) of nonsporulated preculture (OD 600_nm_=2 on various nutritional medium) and incubated at 27°C and 180 rpm with D-glucose and straw (Worthy of note here is that straw was taken as the biochemistry differs from straw to straw.) in 1 L bottles. Growth was tracked by monitoring light scattering at 600_nm_ with a SmartSpec™ 3000 spectrophotometer over a period of 72 h. Growth kinetics experiments were determined on a graph representing Ln (OD 600 nm)= f(t). Doubling times (d) were calculated during the exponential phase according to the formula: n= (Ln(ODt_2_)-Ln(ODt_1_))/Ln(2) and d=t_2_-t_1_/n where n represents the number of generations. Cultivation performance was in general judged by the yield of the CX production. As units, the yield per volume of cultivation broth (g 1000 m L^-1^) and specific yield per biomass cell weight g 1000 m L^-1^ were measured at the end of cultivation. For determination of specific productivity the growth curve of the *D. natronolimnaea* svgcc1.2736 strains, using BDW, as biomass was integrated, yielding the biomass dry weight integral (BDWI).

(6) $$\begin{array}{l} \mathrm{BDW}I_{t2}=\mathrm{BDW}I_{t1}\\ \;+\left[\frac{\mathrm{BD}W_{t1}+\mathrm{BD}W_{t2}}{2}•\left(t_{2}-t_{1}\right)\right] \end{array}$$

For biomass dry weight was determined following the protocol given by Wucherpfennig (2011) with medications. Culture samples (10 mL) were taken in 20-mL centrifuge tubes. The cells were measured gravimetrically by filtering (Nalgene 300–4100) a defined amount of biomass suspension through a predried and pre-weighted suction filter (Filter Paper, Grade 392, Anugrah Niaga Mandiri) and dried at 105°C to a constant weig for 48 h. Prior to drying (105°C at 48 h), the filter was rinsed several times with deionized water to remove medium components from the biomass [77]. The biomass dry weight concentration (g 1000 m L^-1^) was calculated as the difference between the weight of the filter with and without dried biomass divided by the sample volume.

### CX extraction and analysis

Extraction of the CX was done following the method described previously by Asker (1999) with modifications; 10 mL aliquots of cultures were centrifuged at 7,000 g (3–6°C) for 20 min using a cooling centrifuge (Eppendorf, 5427 R). The cell pellets were washed twice with deionized water (NaCl; 9 g L^-1^) and centrifuged again. These cells were resuspended three times in 6 ml of methanol by repeated centrifugation for 18 min until the cell debris turned colorless and transferred to hexane (HPLC Waters Acquity 2996 PDA) [78]. The CX extracts were subsequently filtered through a 0.45 μm hydrophobic PTFE membrane (Waters) and analyzed by scanning the absorbance in the wavelength region of 350–650 nm using the UV–Vis spectrophotometer (U-2800, Hitachi). The maximum absorbance was determined at a wavelength of 474 nm=λ _max_. The results are given as CX yield (mg)/1,000 mL of culture. Chromatographic separation was performed on a reverse-phase C18 column (250 mm×4.6 mm, Waters) where the temperature of the column was maintained at room temperature. The mobile phase used was a mixture of methanol and acetonitrile (20:80, V/V) at a flow rate of 1 mL min^-1^. The pressure was 1.05 kpsi and the injection volume was 20 μL. The peaks were evaluated based on their absorbance at 474 nm. Retention time and concentration of the samples were compared with pure standards of CX (Sigma-Aldrich, USA). CX amount was calculated by using the formula recommended by Schiedt (1995) [79].

(7) $$\mathrm{CX}\left(\mathrm{mg}/1000\;\mathrm{mL}\right)=\frac{A_{474}\times V_{S}\times 10^{9}}{A_{1\mathrm{cm}}^{1\%}\times 100}$$

Where A_474_, V_s_ and $A_{1\mathrm{cm}}^{1\%}$ are the absorbance maximum of CX in methanol the volume of sample solution, and the specific absorption coefficient of CX for a 1% solution in a 1cm cell (in methanol, $A_{1\mathrm{cm}}^{1\%}=2200$), respectively.

### Validation experiments by RSM

RSM was used to validate the effect of biomass and CX production by the *D. natronolimnaea* svgcc1.2736 strains mutant. The effects of four process parameters (considered as independent variables) namely D-glucose content (12.5-25 g L^-1^), Mg^2+^ concentration (15–40 ppm), mannose content(6.75-25 g L^-1^) and irradiation dose (0.5-4.5 Gy) on the BDW and CX yield were studied 30 treatments were conducted based on the CCD, each at three coded levels −1.25, 0 and +1.25. Experiments were randomized in order to minimize the effects of unexplained variability in the observed responses due to extraneous factors [80]. Experiments were randomized in order to minimize the effects of unexplained variability in the observed responses due to extraneous factors. Our preliminary studies showed that the addition of the concentration levels studied to the culture medium resulted in desirable amounts of CX and BDW by the mutant strain. For statistical calculations, the relation between the coded values and actual values are described by Equation (8). The coded values of the process parameters were determined by the following as under:

(8) $$x_{i}=\left(X_{i}-\overset{-}{X_{i}}\right)/\left(\Delta X_{j}/2\right)\left(i=1,2,3,\ldots ,k\right)$$

Where *X*_*i*_ is dimensionless value of an independent variable, *X*_*i*_ is real value of an independent variable, $\overset{-}{X_{i}}$ is real value of the independent variable at the central point and *ΔX*_*j*_ is step change.

A mathematical model, relating the relationships among the process dependent variable and the independent variables in a second-order equation, was developed. The regression analysis was performed to estimate the response function as a second order polynomial. The model equation for analysis is as under:

(9) $$\begin{array}{l} Y_{i}=\varpi_{0}+\sum_{i=1}^{k}\varpi_{i}X_{i}+\sum_{i=1}^{k}\varpi_{\mathrm{ii}}X_{i}^{2}\\ \;+\sum_{i=1,≺j}^{k-1}\sum_{j=2}^{k}\varpi_{\mathrm{ij}}X_{i}X_{j} \end{array}$$

Where *Y*_*i*_ is the response value, *X*_*i*_ are the coded values of the factors, *ϖ*_0_ is a constant coefficient, *ϖ*_*i*_ are the linear coefficients, *ϖ*_*ii*_ are the quadratic coefficients and *ϖ*_*ij*_(i and j) are the interaction coefficients [81]. The statistical software package SPSS 20 was used for regression analysis of the data obtained and to estimate the coefficient of the regression equation. The equations were validated by the statistical tests called the ANOVA analysis. The optimal values of the test variables were obtained in coded values and transformed to uncoded values. To establish the individual and interactive effects of the test variable on the CX production response surfaces were drawn.

## Abbreviations

BDW: Biomass dry weight; BDWI: Biomass dry weight integral; CX: Canthaxanthin; AT medium: Peptic digest of animal tissue 5.000 Gms Litre^-1^; Yeast extract 3.000 Gms Litre^-1^; Malt extract 3.000 Gms Litre^-1^; Dextrose 10.000 Gms Litre^-1^; Agar 20.000 Gms Litre^-1^; HIRFL: Heavy ion research facility in lanzhou; RBE: Relative biological effectiveness; RSM: Response surface methodology; LET: Linear energy transfer; MKM: Microdosimetric kinetic model; SOBP: Spread-out Bragg peak; S(D): Surviving fraction; CT: Carotenoid; HPLC: High performance liquid chromatography; CCD: Central composite design; ANOVA: Analyzed by analysis of variance; TST: Transportation safety technologies.

## Competing interests

The authors declare that they have no competing interests.

## Authors’ contributions

XZ carried out the research work and conceived and organized the study and drafted the manuscript. JRX carried out the CX yield measurement and residues composition analysis, and participated in the drafting of the manuscript participated drafted the manuscript. LT was involved in revising the manuscript critically for important intellectual contents. ZJX was involved in data verification and designed the optimization experiment. FWZ contributed in data interpretation. XHL carried out growth and CX production studies. MRZ helped in some experimental work. WL helped in some experimental work. JPL helped to analyze results and to draft the manuscript. All authors read and approved the submitted version of manuscript.
